# Adjustment to Subtle Time Constraints and Power Law Learning in Rapid Serial Visual Presentation

**DOI:** 10.3389/fpsyg.2015.01748

**Published:** 2015-11-18

**Authors:** Jacqueline C. Shin, Seah Chang, Yang Seok Cho

**Affiliations:** ^1^Skill and Coordination Laboratory, Department of Psychology, Indiana State UniversityTerre Haute, IN, USA; ^2^Human Performance Laboratory, Department of Psychology, Korea UniversitySeoul, South Korea

**Keywords:** rapid serial visual presentation, attentional blink, skill acquisition, temporal learning, implicit learning

## Abstract

We investigated whether attention could be modulated through the implicit learning of temporal information in a rapid serial visual presentation (RSVP) task. Participants identified two target letters among numeral distractors. The stimulus-onset asynchrony immediately following the first target (SOA1) varied at three levels (70, 98, and 126 ms) randomly between trials or fixed within blocks of trials. Practice over 3 consecutive days resulted in a continuous improvement in the identification rate for both targets and attenuation of the *attentional blink* (AB), a decrement in target (T2) identification when presented 200–400 ms after another target (T1). Blocked SOA1s led to a faster rate of improvement in RSVP performance and more target order reversals relative to random SOA1s, suggesting that the implicit learning of SOA1 positively affected performance. The results also reveal “power law” learning curves for individual target identification as well as the reduction in the AB decrement. These learning curves reflect the spontaneous emergence of skill through subtle attentional modulations rather than general attentional distribution. Together, the results indicate that implicit temporal learning could improve high level and rapid cognitive processing and highlights the sensitivity and adaptability of the attentional system to subtle constraints in stimulus timing.

## Introduction

In order to navigate a dynamic environment, humans and other animals must attend selectively to goal-relevant perceptual information. Fortunately, meaningful environmental information is often patterned and predictable, making is possible for the information about spatial layout and timing of critical information to be learned and used to guide attention. While previous research illuminates the anticipatory and learning processes associated with attending in the spatial domain, processes that allow us to modulate attention in the time domain are less clear. Can humans use past experience to allocate attention to specific moments in time for perceptual and cognitive analysis? The goal of our study was to investigate whether the timing of stimuli could be implicitly learned to enhance attentional selection.

Previous research indicates that attention can be modulated in space and time effectively with the use of cues. In the spatial domain, pre-cuing allows focused attention to be guided to specific locations (Posner et al., 1980). In the temporal domain, knowledge of the fore-period between a stimulus cue and a target stimulus can be used to improve visual stimulus detection (Coull and Nobre, 1998), auditory reaction time (Karlin, 1959), and visual orientation discrimination (Westheimer and Ley, 1996; Grosjean et al., 2001). Thus, advance knowledge of stimulus timing can facilitate performance by guiding the allocation of attentional resources to the moment of stimulus presentation. A joint spatial-temporal representation might underlie temporal anticipation effects, since pre-specified fore-periods can amplify spatial cuing effects (Kristjánsson et al., 2010).

It is less clear whether spatial or temporal information acquired through implicit learning can be used to modulate attention. Spatial and temporal patterns can be learned implicitly to facilitate motor actions. Practice with a repeating spatial pattern in a serial reaction time task can lead to faster motor responses relative to when such a pattern is absent (Nissen and Bullemer, 1987). Such a benefit is found even when participants are not aware of the spatial pattern. Similarly, repeated exposure to a temporal pattern can also facilitate faster responding without conscious awareness of the pattern in the same type of task (Shin and Ivry, 2002). However, due to the large motoric component in serial reaction time studies, it remains ambiguous whether the sequence benefit reflects learning related to attentional processes or to motor planning. The use of an alternative paradigm, the visual search task, speaks more straightforwardly to implicit learning that involves attentional modulation. Consistent presentation of spatial stimulus configurations in a visual search task can speed up target detection, pointing to implicit learning that influences the spatial control of attention (Chun and Jiang, 1998). In the temporal domain, a temporal pattern of serially presented stimuli can be learned as an effective contextual cue that predicts the likelihood of subsequent target presentation (Olson and Chun, 2001). However, evidence is lacking for the dynamic adjustment of attention to implicitly learned temporal patterns themselves within the visual search paradigm.

The rapid serial visual presentation (RSVP) paradigm has been used extensively to study how attention is deployed over time at sub-second time scales. Of particular interest is the *attentional blink* (AB) phenomenon, where the probability of identifying or detecting a target (*T2*) among distractor stimuli changes depending on its distance in time from a preceding target (*T1*) (Raymond et al., 1992; Wyble et al., 2009). Specifically, T2 identification or detection rate declines at target onset asynchronies (*TOA*s) of 200–400 ms and recovers after that point. This non-linear profile may reflect an attentional bottleneck at which stimulus identity is consolidated into an episodic memory representation that allows conscious report (Raymond et al., 1992; Chun and Potter, 1995; Bowman and Wyble, 2007) or some other limitation in central processing resources (Jolicoeur, 1998; Di Lollo et al., 2005; Kawahara et al., 2006; Akyürek et al., 2007a). Alternatively, the AB deficit may be a side effect of excessive control processes that are deployed to protect the identity of targets from interference in memory (Taatgen et al., 2009) or other goal-driven reactions to stimulus categories (Olivers and Meeter, 2008; See Martens and Wyble, 2010 for a review).

Recent RSVP studies investigated whether knowledge about target timing could improve performance. Pre-cues indicating target timing can effectively improve target identification (Martens and Johnson, 2005; Hilkenmeier and Scharlau, 2010; Badcock et al., 2013; Visser et al., 2014). Martens and Johnson (2005, Experiments 2 and 3) found that presenting a cue signaling the TOA before each RSVP trial improved T2 identification during the AB interval, and consequently, led to a smaller AB deficit. Such results indicated that conscious temporal expectations guided by a pre-cue could guide attentional modulation even with fast RSVP displays. Interestingly, positive effects of a temporal cue on performance can manifest within a couple 100 ms of the cue (Hilkenmeier and Scharlau, 2010).

The results are more complex concerning the learning and use of temporal information provided through the consistent presentation of temporal information. On the one hand, many avenues of evidence indicate that *explicit* temporal expectancy can improve RSVP performance and even reduce the magnitude of the AB deficit. Explicit knowledge about target timing that is fixed across trials within a block can improve RSVP performance (Tang et al., 2014; Visser et al., 2014). Visser et al. (2014) found that, compared to when TOA varied unpredictably across trials, performance was better when TOA was fixed on every trial and explicit instructions were given to participants alerting them to this consistency and encouraging them to use that temporal information in performing the task. Similarly, consistency in TOA/lag also benefited performance when attention was drawn to the second target by presenting it in a salient color (Choi et al., 2012; Tang et al., 2014). In Choi et al. (2012), highlighting the second target when it was displayed in a fixed position (Lag 2) led to reduced AB, perhaps due to a general heightening of attention to T2 or a facilitation of temporal expectancies from stimulus salience. In the absence of explicit cues or highlighting of T2, the effect of temporal consistency on performance appears to be variable. In Martens and Johnson (2005, Experiment 1), target identification rates were *not* enhanced even when the TOA was fixed on every trial within a participant group if explicit instructions concerning the predictable TOA were absent. However, it is possible that the amount of practice in this study was insufficient for implicit learning to occur. In contrast, Willems et al. (2015) found that consistent presentation of T2 at a fixed lag manipulated between participants led to enhanced temporal expectancies from temporal regularities. Because the TOA was fixed between participants, any positive effects of temporal consistency likely reflected conscious temporal expectancies.

Relatedly, Lasaponara et al. (2015) and Martin et al. (2011) showed that the degree of uncertainty in the timing of targets in RSVP tasks impacts information processing during an RSVP task. Interestingly, Martin et al. (2011) found that temporal noise enhanced RSVP performance, contrary to expectations based on learning from temporal regularities. Martin et al. (2011) attributed this benefit of temporal noise to a distraction of attention away from T1, attenuating the overinvestment of attention to T1 and the consequent AB.

Whereas the above studies manipulated the cuing or consistency of TOAs in RSVP tasks, Akyürek et al. (2007b, 2008) manipulated stimulus duration while keeping the TOAs constant. Akyürek et al. (2008) found reduced AB and changes to the proportion of target reversal errors when a longer stimulus duration (70 vs. 30 ms) was expected. These changes were attributed to attentional modulations in response to a slower perceived stimulus presentation rate, involving changes in the likelihood that T1 and T2 were integrated into a common episodic representation. Whereas this study did not manipulate the consistency of stimulus duration, it would be informative to directly manipulate the consistency of a temporal parameter independently of TOA/lag in evaluating the role of learning in attentional adjustments.

Recent work demonstrating practice effects on AB and the psychological refractory period (Garner et al., 2014) indicate that consistent aspects of stimulus presentation can make possible attentional modulations, such as those involving allocation of attention between T1 and T2 (Slagter et al., 2007; Nakatani et al., 2012; Willems et al., 2015). AB can be reduced through enhanced salience of T2 (Choi et al., 2012; Tang et al., 2014) or other experimental manipulations designed to draw attention to T2 in the absence of consistency in TOA (Livesey et al., 2009). AB has also been shown to be reduced when the distribution of attention over the course of an RSVP trial was varied through the utilization of concurrent tasks (Olivers and Nieuwenhuis, 2005; Taatgen et al., 2009), videogaming experience (Green and Bavelier, 2003), or meditation training (Slagter et al., 2007). Interestingly, because these studies involve practice over multiple days, they likely reflect sleep induced enhancements in neural mechanisms supporting attentional control (Cellini et al., 2015). Although these studies did not directly manipulate specific timing parameters, it is worth noting that the consistency in the periodic timing of stimuli in the RSVP tasks may also have contributed to enhanced RSVP performance. The idea that periodicity can influence attentional processes appears plausible in the context of oscillatory attentional dynamics, which can be induced through a series of periodically presented stimuli at 2 Hz (Jones et al., 2002) and 10 Hz timescales (Mathewson et al., 2010). However, this type of oscillatory attention is theorized to form through a within-trial process of aligning attentional peaks to stimulus onset times, called *entrainment* (Large and Jones, 1999), rather than learning achieved through practice. Research is lacking as to whether periodicity can lead to performance enhancements in the RSVP task through within-trial entrainment, temporal learning over practice, or both.

Taken together, the above work on temporal cueing and on the effects of temporal consistency indicates that advance temporal information can be used to modulate attention when this information is learned in a conscious and strategic manner. However, evidence is lacking with respect to whether consistency in the timing of stimuli can be learned implicitly in a way that would aid RSVP performance. The primary goal of the current study was to manipulate temporal consistency and investigate whether a consistent time constraint could lead to attentional adjustment through implicit learning in an RSVP task, resulting in improved RSVP performance. Specifically, we manipulated a single temporal parameter in the RSVP display to be blocked at various levels within participants or randomly presented from trial to trial. By utilizing a two-target identification version of the RSVP task, we addressed whether such implicit temporal learning could improve higher level cognitive processing (symbol identification) as opposed to lower level processes (such as category or target detection). Furthermore, we employed procedures designed to maximize the potential for implicit learning of the temporal intervals to occur. Conceptualizing attention in the RSVP task as an internal cognitive skill, we hypothesized that extensive practice with a consistent time constraint would result in the gradual adjustment of attentional dynamics to maximize the temporal selection of both targets.

We expanded upon the design of the Martens and Johnson (2005, Experiment 1) implicit learning experiment in three ways. First, we manipulated timing in a subtle manner to minimize the potential for explicit learning and strategic attentional modulation. Specifically, we manipulated the stimulus-onset asynchrony that immediately followed the display of the first target (SOA1) at three levels in steps of 28 ms—miniscule compared to the difference between 270 and 720 ms in Martens and Johnson (2005). This also allowed the TOA to be manipulated independently of the number of intervening distractors (the lag) and while keeping target duration constant. It is important to note that in all the above studies that manipulated the time interval separating targets (the TOA), this was correlated with the number of distractors separating targets (the *lag*). Thus, it remains unclear whether the attentional adjustments associated with explicit temporal information were driven by anticipation of time intervals or triggered by a contextual cue consisting of distractors. Therefore, in studying the role of implicit temporal learning in an RSVP task, it would be useful to dissociate the effects of implicit temporal learning from cuing effects. We also fixed T1 at the second position in order to minimize the uncertainty of T1 timing and to focus our timing manipulation on T2.

The second modification of the Martens and Johnson (2005) experiment lay in the way the consistency of the to-be-learned time constraint was manipulated. Specifically, SOA1 was blocked for one participant group and randomly presented across individual trials for another participant group. This within-participant variation of the time constraint contrasted with Martens and Johnson (2005) and other studies in which a fixed target timing was implemented between participants. One reason for varying the time constraints within participants was that this was thought to be conducive to implicit learning in light of work in motor skill that shows superior learning and retention for *varied practice*, where multiple levels of a variable are experienced, relative to *constant practice*, where only a single variable level is experienced (Wulf and Schmidt, 1997).

A second reason for the within-participant blocking of SOA1 was to allow for a closer evaluation of the time course of processing for the two targets within a close temporal range. First, based on the findings of Potter et al. (2002), we hypothesized that at Lag 1, the demand to process T2 would interfere with T1 processing and that this interference would be greater with shorter SOA1s. Second, we expected a greater proportion of target order reversal errors for shorter SOA1s. We assumed that reversal errors are caused by the failure to commit information about the identity of both targets to distinct episodic memory traces (Akyürek et al., 2007b; Wyble et al., 2009) and hypothesized that both targets would fall within the same encoding window more frequently with shorter SOA1s. More relevant to the goals of the current study, we predicted that blocked SOA1s would lead to greater reversal errors at early lags than randomly presented SOA1s. This prediction is driven by the hypothesis that consistent timing would result in enhanced processing of T2, which would increase the odds of prior T2 entry into working memory or increase the tendency for T2 to be encoded together with T1 during the same encoding time window. Alternatively, consistent timing could result in changes in the temporal duration of the episodic encoding window. Conceivably, these two mechanisms could both contribute to changes in the proportion of reversal errors.

Last, but not least, we included a notably greater number of practice trials relative to previous studies in an effort to maximize our chances of observing effects of consistency in the subtle time constraints described above. Specifically, we had participants practice over three consecutive days that each included 540 RSVP trials (including 54 practice trials). Importantly, we were able to more fully observe the improvement in target identification and reduction in AB as a function of practice. Although positive effects of practice on RSVP performance have already been documented, the time course of such effects have not been explored in detail over such an extended period of practice. In a vast number of studies on perceptual-motor as well as cognitive skills, performance improves as a negatively accelerating function of practice, where greater gains are found in the earlier, relative to later, phases of practice (Newell and Rosenbloom, 1981). This characteristic of learning curves is often referred to as the “power law” of skill acquisition in recognition of the wide range of skills that exhibit this feature (Newell and Rosenbloom, 1981). To the extent that attentional selection in the RSVP task is a skill that can be developed through similar learning processes as other cognitive or perceptual-motor skills, we expected target identification rates and the magnitude of the AB would show learning curves that followed a negatively accelerating curve as a function of practice.

## Methods

### Participants

Forty-nine students at Korea University, South Korea participated for the equivalent of about 30 US dollars—25 in the random condition and 24 in the blocked condition. These participants were recruited and tested with the approval of the Institutional Review Board at Korea University (KU-IRB-13-31-A-2).

### Procedure

**Figure 1:**
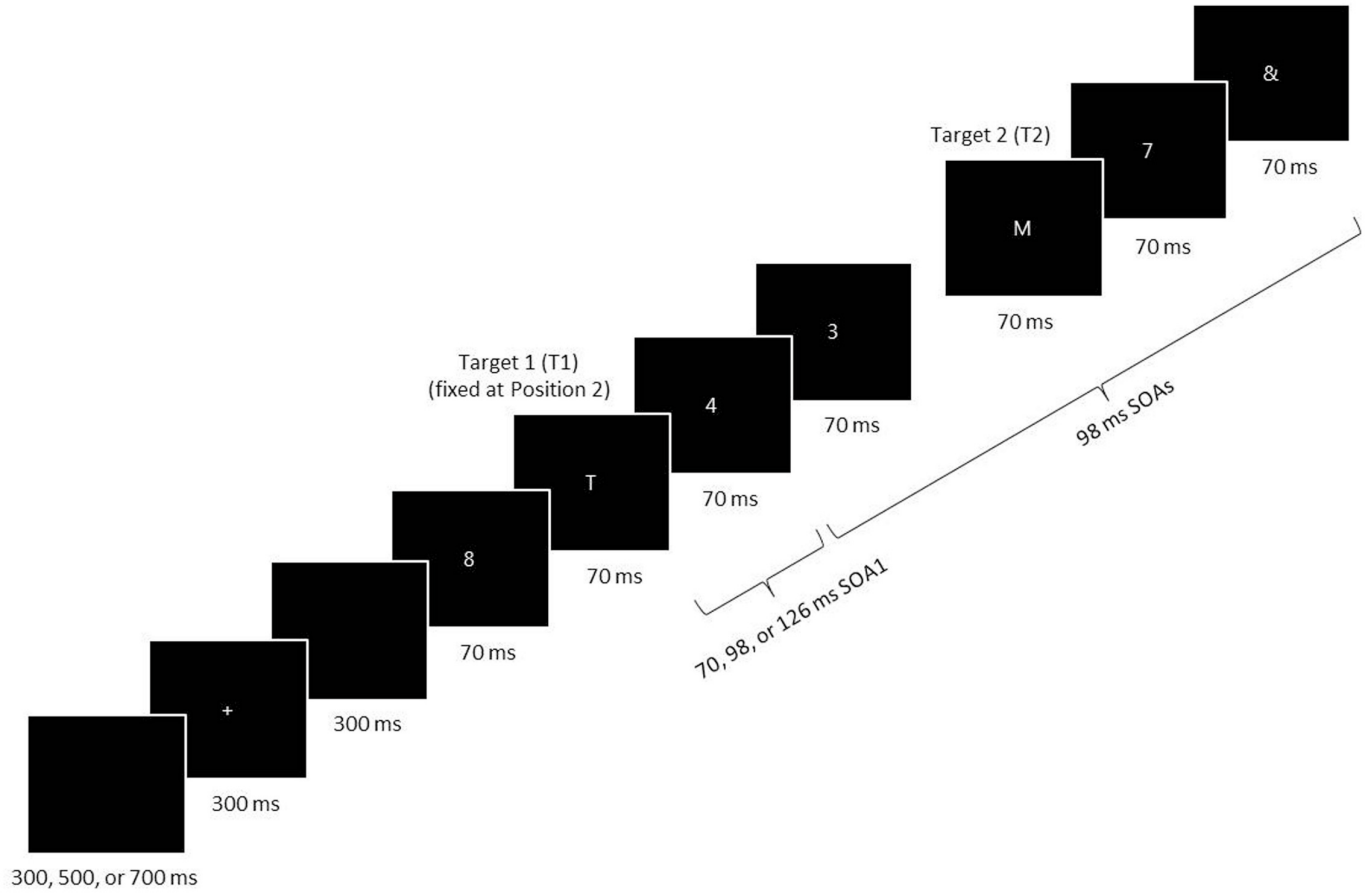
**A schematic of an RSVP trial**. Two target letters (T1 and T2) were displayed among a stream of distractor numerals. The last stimulus was an & mask. T1 was always presented at the second position in the RSVP series, and T2 followed T1 with a lag of 1–6. The stimulus-onset asynchrony (*SOA*) was always 98 ms, with the exception of the first SOA immediately following T1 (*SOA1*), which was short (70 ms), middle (98 ms), or long (126 ms). SOA1 varied among trials (in the *random condition*) or were fixed within a block (in the *blocked condition*). All targets and distractors were displayed for 70 ms before disappearing from the computer screen.

Participants performed an RSVP task in three experimental sessions that took place over consecutive days. Figure 1 illustrates the stimulus events during a single RSVP trial, programmed and presented on a PC using the *Pascal* programming language. White stimuli were presented one at a time on a black background in the center of a CRT screen. The background luminance was 1.96 cd/m^2^. The participant began each trial by pressing the spacebar. After a random delay of 300, 500, or 700 ms, a fixation cross was displayed for 300 ms. Then, after a 300 ms delay, a stimulus series was presented consisting of two letter targets, seven numeral distractors, and an & mask at the end of the series. The stimuli, 5 × 3 mm (0.48° × 0.29° in visual angle) blocked characters, were viewed at a distance of ~60 cm. The targets consisted of capitalized letters taken from the alphabet, excluding *I, L, O, U*, and *V*, and the two targets on a given trial were never identical. The distractors consisted of numerals selected randomly from 2 to 9 with the caveat that successive distractors on a given trial were never the same.

On every trial, the first stimulus in the series was a distractor, and the first target (*T1*) was presented in the second position in the series. The second target (*T2*) was presented after a lag of 1–6, that is, with 0–5 distractors intervening between the two targets. The penultimate stimulus was always a distractor, and this was directly followed by the & mask. After the RSVP stimulus series was displayed, the participant keyed in the targets in the remembered order of presentation using the computer keyboard. (S)he was permitted to amend responses. If the participant failed to remember a target or the order of targets, (s)he was encouraged to guess. Responses were neither timed nor speeded. Feedback was not given with respect to the accuracy of responses.

Stimulus durations and inter-stimulus intervals (*ISI*s) were varied as multiples of the 14 ms computer screen refresh cycle. Each stimulus in the series was displayed for a duration of 70 ms. The ISI was 28 ms, except after the display of T1, when it was 0, 28, or 56 ms. Thus, the first stimulus-onset asynchrony following T1 (*SOA1*) was 70 ms (the *short SOA1*), 98 ms (the *middle SOA1*), or 126 ms (the *long SOA1*).

In each of the three experimental sessions, the participant was presented with nine blocks of 54 trials divided into sets of three blocks each. Thus, there were a total of nine sets of blocks in the whole experiment. The central manipulation in this study was the consistency of SOA1 presentation across trials in a block. For half the participants—in the *random* condition—SOA1 was randomly determined from trial to trial throughout the whole experiment. The number of trials with each of the three SOA1s was equated within each set of blocks, as was the number of trials for each lag. For the other half of the participants—in the *blocked* condition—SOA1 remained the same on each trial within a block but differed across blocks. Specifically, each SOA1 was presented for exactly one block in each set of three blocks. The order of blocks was counterbalanced across sets of three blocks for each participant and across participants. In addition, for a given participant, block order was held constant over experimental sessions. The number of trials with each lag was equated within each block.

Each experimental session began with a practice block of 54 trials that reflected the organization of trials and blocks in the experimental blocks. That is, in the blocked condition, this practice block consisted of three groups of 18 trials, each with one of the three SOA1s and three trials with each of the six lags. In contrast, in the random condition, SOA1 and lag were presented in a random order during the practice block.

Participants were not queried about their awareness of the timing manipulations. None of the participants spontaneously reported any awareness of temporal patterns or irregularities either during or after performing the RSVP trials.

The participant self-paced the entire procedure and performed the task alone after the practice block. The participant took brief breaks between blocks, and there was a forced 60 s break between sets of three blocks, during which the experimenter checked the progress of the experiment. For each experimental session, the entire process took approximately 1 h and 10 min.

## Results

Our analysis focused on three aspects of performance. First, we tested whether practice led to improvements in target identification and examined the shape of the learning curves. Second, we examined whether the AB was reduced with practice and how the SOA1 timing manipulation influenced AB. Third, we investigated whether temporal learning influenced performance at early lags, where SOA1 was expected to have immediate impact on performance.

We report the results concerning these aspects of performance from 47 (23 in the random condition and 24 in the blocked condition) of the 49 participants. That is, the data from two participants in the random condition were excluded from further analysis whose mean proportion correct T1 identification (*p*(T1)) or proportion correct T2 identification conditional on correct T1 identification (*p*(T2|T1)) in Session 1 was below the group mean minus one standard deviation (0.66 for *p*(T1) and 0.40 for *p*(T2|T1)). A target was considered to be identified correctly if its identity was reported regardless of order of report.

### General effects of practice on target identification

**Figure 2:**
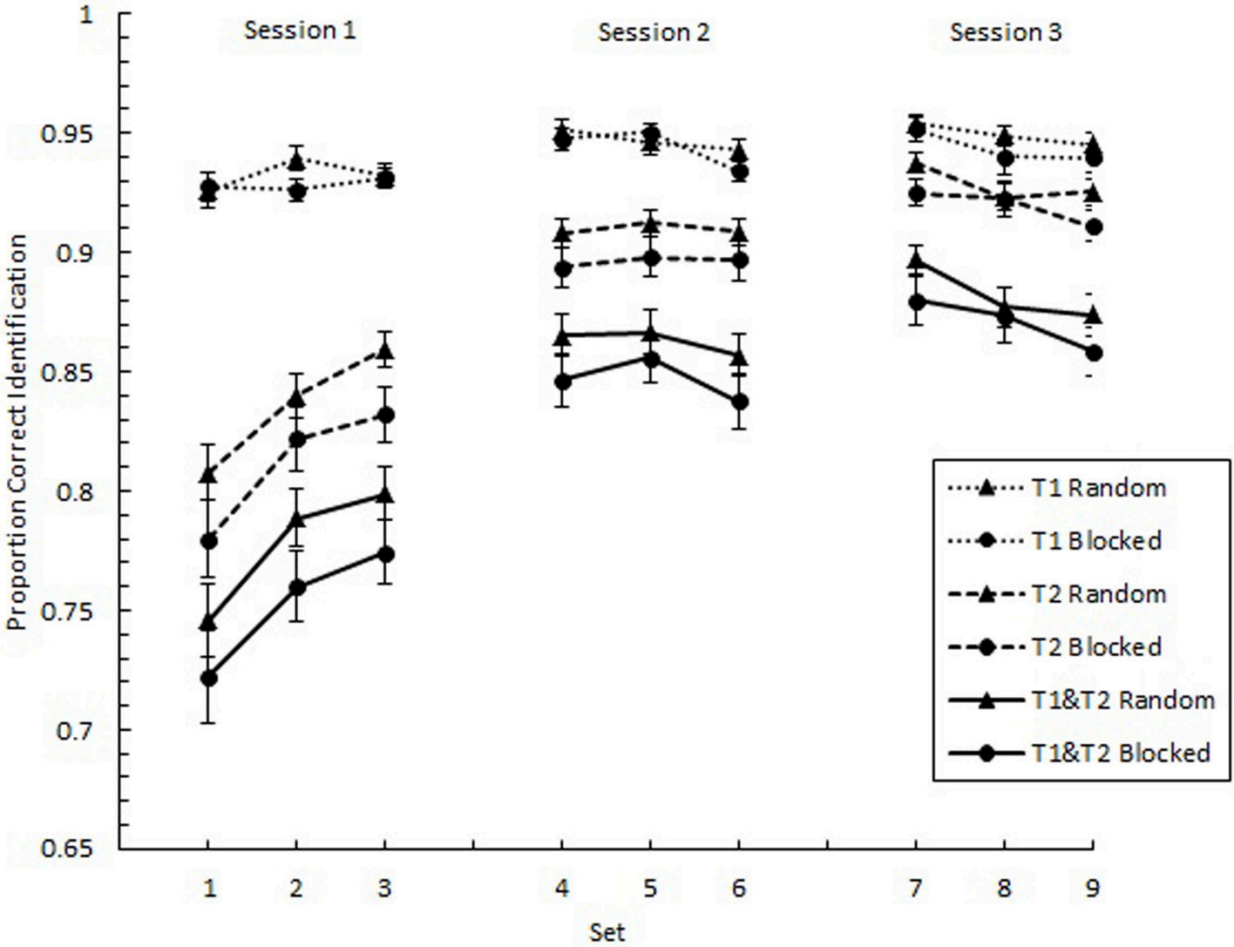
**Proportion correct identification plotted as a function of Set for the first target (T1) and the second target (T2) and for the simultaneous identification of both T1 and T2**. Each session, consisting of three Sets, was administered on consecutive days. Each Set contained three blocks of 54 trials each. Triangles represent performance in the random condition, and circles represent performance in the blocked condition. Error bars represent standard errors.

We analyzed the overall effects of practice with respect to proportion correct identification of individual targets (*p*(T1) and *p*(T2)) as well as the proportion of trials on which both targets were identified correctly (*p*(T1&T2)). We expected to find negatively accelerating learning curves, where greater gains are found in the earlier than later parts of practice. In Figure 2, *p*(T1), *p*(T2), and *p*(T1&T2) are plotted as a function of Set across the three experimental sessions separately for the random and blocked conditions. As predicted, these plots show negatively accelerating learning curves. A 9 (Set) × 2 (SOA1 Consistency: random vs. blocked) mixed factors analysis of variance (*ANOVA*) revealed a main effect of Set for all three measures, *F*_(8, 360)_ = 3.92, *p* < 0.001 for *p*(T1), *F*_(8, 360)_ = 60.84, *p* < 0.0001 for *p*(T2), and *F*_(8, 360)_ = 51.60, *p* < 0.0001 for *p*(T1&T2). However, no significant effects of SOA1 Consistency were found, *p*s > 0.4.

### Specific effects of practice on the AB curve

**Figure 3:**
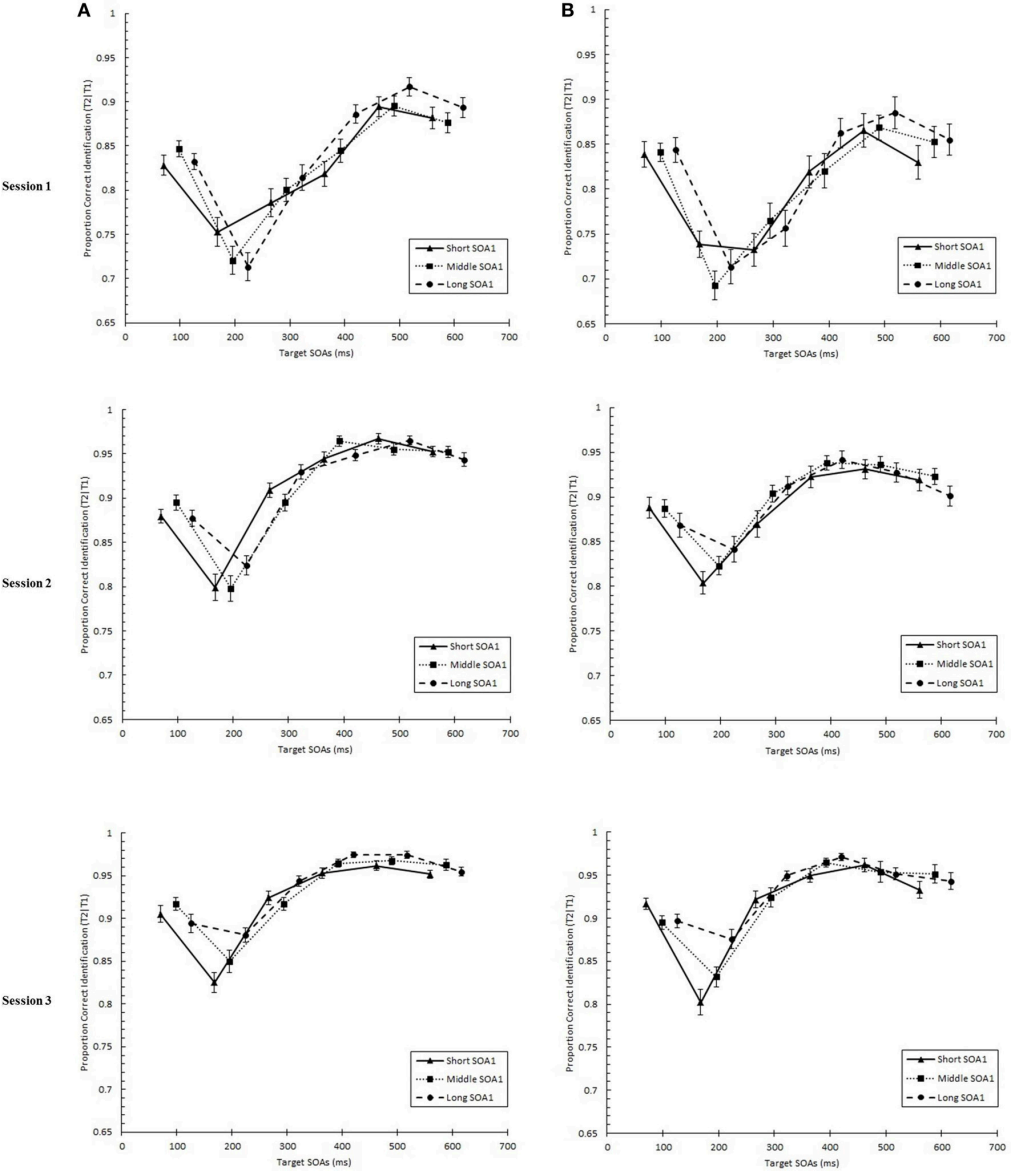
**Proportion correct second target identification conditional on correct first target identification (***p***(T2|T1)) plotted as a function of target onset asynchrony (TOA) for each Session for the random (A) and blocked conditions (B)**. *p*(T2|T1) is plotted separately for the short SOA1 (70 ms, triangles), middle SOA1 (98 ms, squares), and long SOA1 (126 ms, circles).

**Figure 4:**
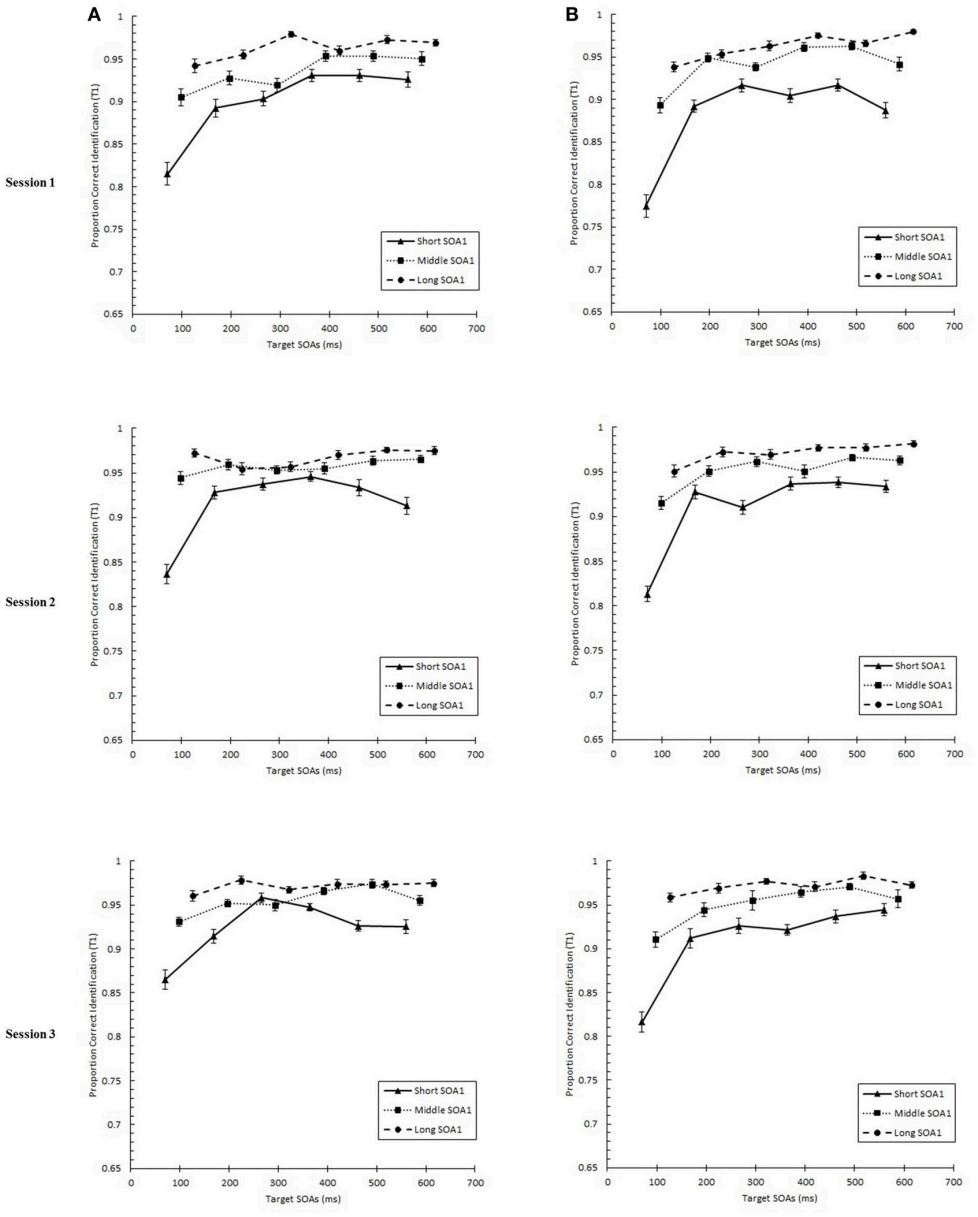
**Proportion correct first target identification (***p***(T1)) plotted as a function of target onset asynchrony (TOA) for each Session for the random (A) and blocked conditions (B)**. *p*(T1) is plotted separately for the short SOA1 (70 ms, triangles), middle SOA1 (98 ms, squares), and long SOA1 (126 ms, circles).

We tested whether practice led to the attenuation of the AB deficit. The AB curve is plotted for each SOA1 [short (70 ms), middle (98 ms), and long (126 ms)] and Session for the random and blocked conditions in Figure 3. Here, *p*(T2|T1) is plotted as a function of target onset asynchronies (TOA). The corresponding graphs are plotted for *p*(T1) in Figure 4. A 3 (Session) × 3 (SOA1) × 2 (SOA1 Consistency) × 6 (Lag) ANOVA was conducted on both measures. The results are reported in the first two subsections below.

#### Presence of AB and effects of SOA1

First, we sought to confirm the presence of the standard AB curve and to determine the effect of SOA1 on the AB curve. We predicted that the identification of both targets would be negatively affected by shorter SOA1s due to an increased potential for competition between T1 and T2 for perceptual and memory processing resources. In addition, T1 would be susceptible to greater backward masking with shorter SOA1s. These processes would manifest as greater ABs (Ouimet and Jolicoeur, 2007; Visser, 2007). Finally, we were interested in how the potential to learn the SOA1s, that is, SOA1 Consistency, influenced the AB.

We found a standard AB curve, as indicated by a main effect of Lag, *F*_(5, 225)_ = 70.00, *p* < 0.0001, but the shape of the AB curve differed slightly for the blocked and random conditions, *F*_(5, 225)_ = 3.72, *p* < 0.01. Paired comparisons showed that while *p*(T2|T1) was lower at Lag 2 than any of the other lags in both conditions, the recovery phase (Lags 4–6) was greater than Lag 1 sparing and Lag 3 only for the random condition but not for the blocked condition.

With respect to the effects of SOA1, *p*(T2|T1) was greater the longer the SOA1, *F*_(2, 90)_ = 10.09, *p* < 0.0001. The SOA1 × Lag interaction was also significant, *F*_(10, 450)_ = 2.73, *p* < 0.01, reflecting significant SOA1 effects at AB lags, Lags 2–4, *F*s > 4, *p*s < 0.05. No interactions subsuming both SOA1 and SOA1 Consistency were significant, *p*s > 0.8.

With respect to *p*(T1), a main effect of Lag was found, *F*_(5, 225)_ = 76.54, *p* < 0.0001, reflecting worse performance at earlier lags. This effect of Lag was more pronounced in the blocked than in the random condition, *F*_(5, 225)_ = 3.62, *p* < 0.01. As with *p*(T2|T1), *p*(T1) was greater with longer SOA1s, *F*_(2, 90)_ = 123.62, *p* < 0.0001. Effects of SOA1 were greater at the early lags, *F*_(10, 245)_ = 25.09, *p* < 0.0001, where the effects of backward masking and competition with T2 were expected to be greatest.

These results are consistent with the expectation that shorter SOA1s would lead to worse AB due to increased competition between T1 and T2 and backward masking of T1.

#### Effects of practice on AB

As expected based on the results concerning the general effects of practice, *p*(T2|T1) and *p*(T1) improved over sessions, *p*s < 0.001. In addition, a significant Session × SOA1 Consistency interaction was found for *p*(T2|T1), *F*_(2, 90)_ = 4.30, *p* < 0.05, which reflected a greater rate of improvement in performance for the blocked condition than the random condition.

The shape of the AB changed with practice as evidenced by a significant Session × Lag interaction, *F*_(10, 450)_ = 9.56, *p* < 0.0001. In addition, these practice related changes in the AB curve differed between the blocked and random conditions, *F*_(10, 450)_ = 1.92, *p* < 0.05 for the Session × SOA1 Consistency × Lag interaction.

**Figure 5:**
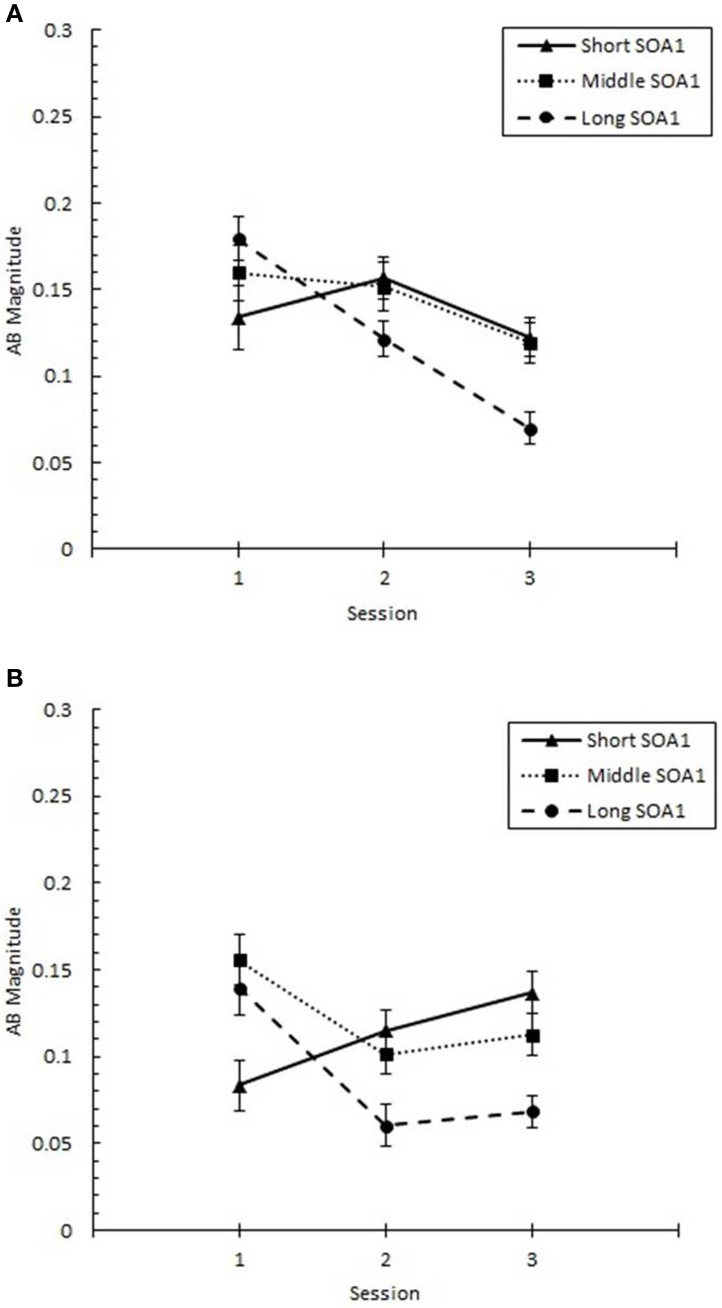
**AB Magnitude plotted as a function of Session for the random (A) and blocked conditions (B)**. AB Magnitude was computed as proportion correct second target identification conditional on correct first target identification (*p*(T2|T1)) at Lag 6 minus *p*(T2|T1) at Lag 2. AB Magnitude is plotted separately for the short SOA1 (70 ms, triangles), middle SOA1 (98 ms, squares), and long SOA1 (126 ms, circles).

These changes in the AB curve included changes in the magnitude of the AB deficit. To more closely examine the effects of practice on the magnitude of AB, we analyzed *AB Magnitude*, defined as *p*(T2|T1) at Lag 6 minus *p*(T2|T1) at Lag 2 and plotted it as a function of Session for each SOA1 in Figure 5. We note that other measures of AB magnitude, such as *p*(T2|T1) at Lag 5 minus Lag 2, yielded a similar pattern of results. A 3 (Session) × 3 (SOA1) × 2 (SOA1 Consistency) ANOVA on AB Magnitude revealed a significant decline with practice, *F*_(2, 90)_ = 4.37, *p* < 0.01, as well as a lower AB Magnitude in the blocked relative to the random condition, *F*_(1, 45)_ = 8.64, *p* < 0.01.

There was a significant Session × SOA1 interaction on AB Magnitude, *F*_(4, 180)_ = 4.90, *p* < 0.001. Whereas AB Magnitude was smaller for the short SOA1 trials than the longer SOA1 trials during Session 1, a reversed trend was apparent by Session 2 due to substantial reductions in AB Magnitude only for longer SOA1s. This is consistent with the interpretation that the backward masking with the short SOA1 increased T1 identification difficulty constraining the lower limit of AB. No other effects on AB Magnitude were statistically reliable.

With respect to *p*(T1), the negative effect of shorter SOA1s on *p*(T1) reported above diminished with practice, *F*_(4, 180)_ = 3.07, *p* < 0.05. No other effects involving Session were significant, *p*s > 0.1.

In sum, the AB was substantially reduced with practice for longer SOA1s, and this reduction was facilitated by consistent SOA1s in the blocked condition.

#### Locus of practice effects on AB

**Figure 6:**
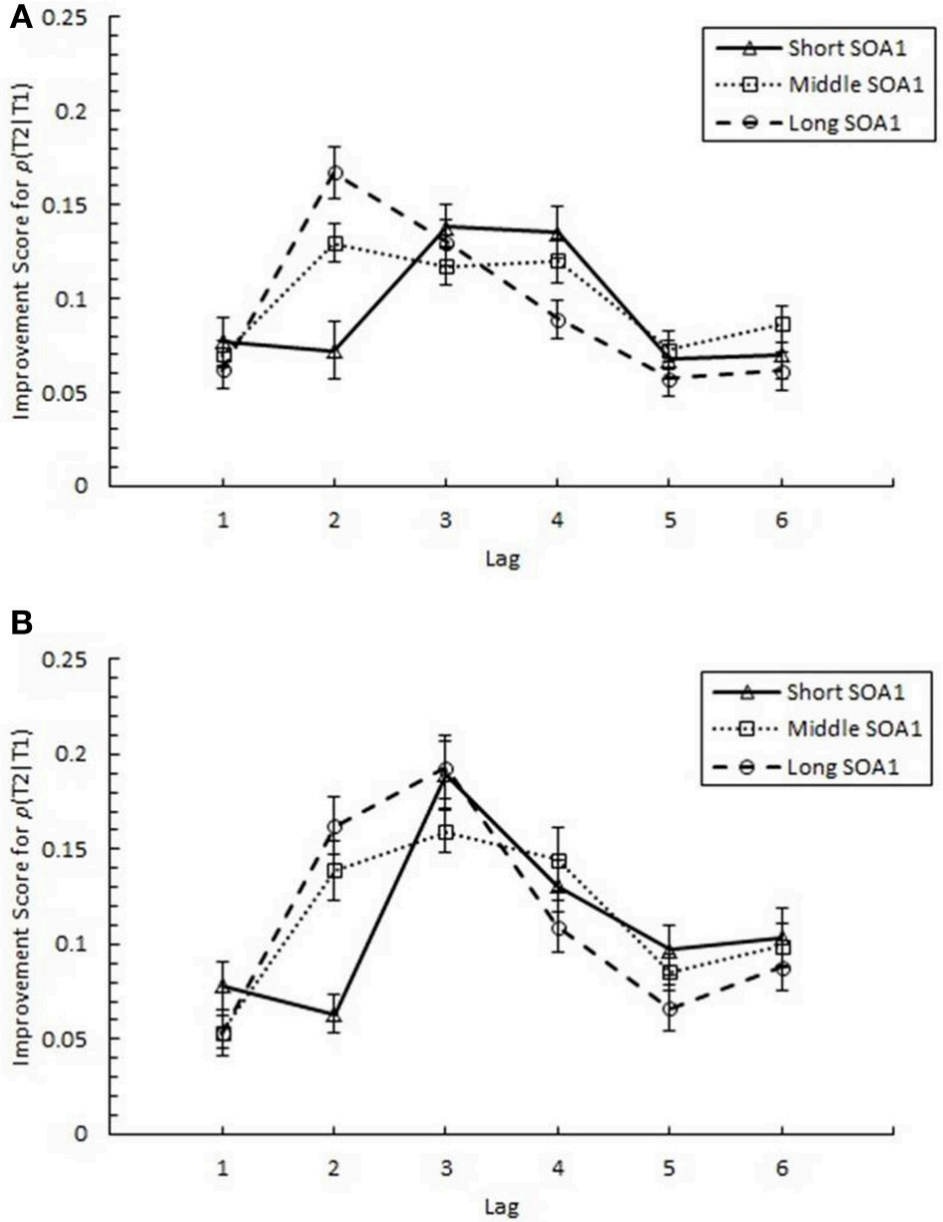
**Improvement Scores (IS) for proportion correct second target identification conditional on correct first target identification (***p***(T2|T1)) as a function of Lag for the random (A) and blocked conditions (B)**. IS for *p*(T2|T1) was computed as *p*(T2|T1) in Session 3 minus *p*(T2|T1) in Session 1. IS for *p*(T2|T1) is plotted separately for the short SOA1 (70 ms, triangles), middle SOA1 (98 ms, squares), and long SOA1 (126 ms, circles).

The above results clearly indicate a reduction in AB with practice. However, it was important to discriminate whether these results were due to increasing *p*(T2|T1) at the AB intervals, as we expected, or to a decrement in recovery from AB at later lags. Therefore, we sought to quantify the degree of practice-related improvement as it occurred across the AB curve. Specifically, we analyzed an *improvement score* (*IS*) for *p*(T2|T1), computed as *p*(T2|T1) in Session 3 minus *p*(T2|T1) in Session 1. IS for *p*(T2|T1) is plotted in Figure 6 as a function of Lag and SOA1. A 6 (Lag) × 3 (SOA1) × 2 (SOA1 Consistency) ANOVA on these scores revealed a significant main effect of Lag, *F*_(5, 225)_ = 13.36, *p* < 0.0001; the greatest improvements occurred at the AB intervals, Lags 2–4 (*M* = 0.134), and the improvements were less at Lag 1 (*M* = 0.069) and Lags 5 and 6 (*M* = 0.081). Consistent with the greater reductions in AB Magnitude with longer SOA1s reported above, IS for *p*(T2|T1) was greater for longer SOA1 only during AB; a Lag × SOA1 interaction, *F*_(10, 450)_ = 3.55, *p* < 0.001, reflected effects of SOA1 only at Lag 2, *F*_(2, 90)_ = 7.46, *p* < 0.005, but not at the other Lags, *F*s < 3, *p*s > 0.09. No effects subsuming SOA1 Consistency reached significance, *p*s > 0.4.

**Figure 7:**
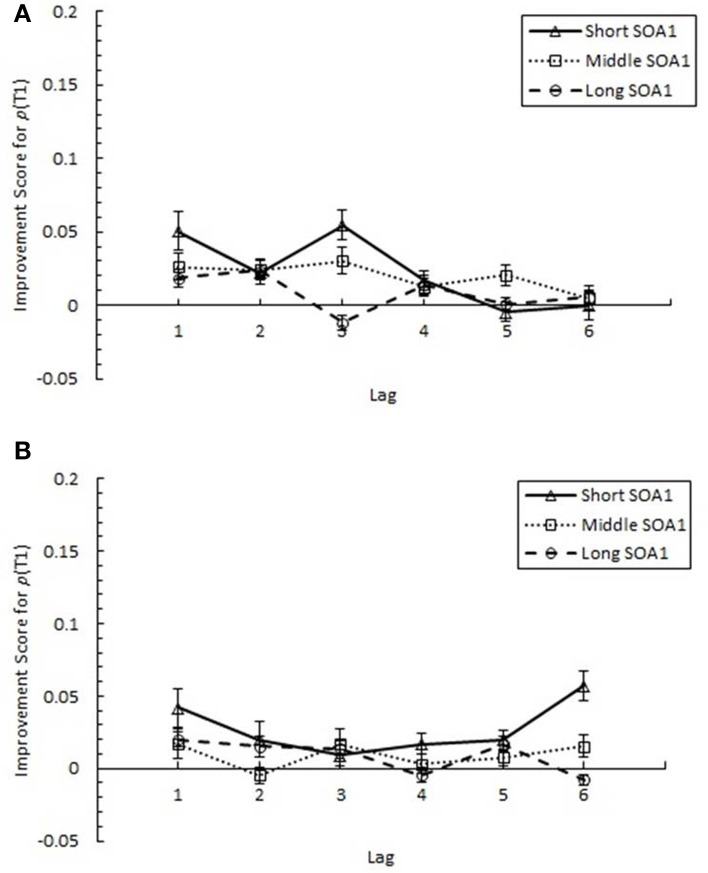
**Improvement Scores (IS) for proportion correct first target identification (***p***(T1)) as a function of Lag for the random (A) and blocked conditions (B)**. IS for *p*(T1) was computed as *p*(T1) in Session 3 minus *p*(T1) in Session 1. IS for *p*(T1) is plotted separately for the short SOA1 (70 ms, triangles), middle SOA1 (98 ms, squares), and long SOA1 (126 ms, circles).

AB reduction did not result from trade-offs involving T1 processing. IS for *p*(T1), plotted in Figure 7, was minimal and did not differ among lags. Interestingly, IS for *p*(T1) was greater with *shorter* SOA1s, *F*_(2, 90)_ = 4.99, *p* < 0.01, signifying a reduction in the negative effects of shorter SOA1s on *p*(T1) with practice. Again, no effects subsuming SOA1 Consistency were significant, *p*s > 0.1.

### Performance at early lags

Effects of temporal learning would most likely manifest at early lags, where shorter SOA1s could result in maximal competition between T1 and T2 processing. We expected that T2 identification and the proportion of target order reversal errors would be greater at shorter SOA1s, whereas T1 identification might show the opposite pattern. Also, if practice and temporal consistency improved the attentional selection of T2 or increased the chances for integrated encoding of the two targets (Akyürek et al., 2007b, 2008), T2 identification and reversal errors at Lag 1 should increase with practice and be greater in the blocked than in the random condition.

#### Target identification at lag 1

**Figure 8:**
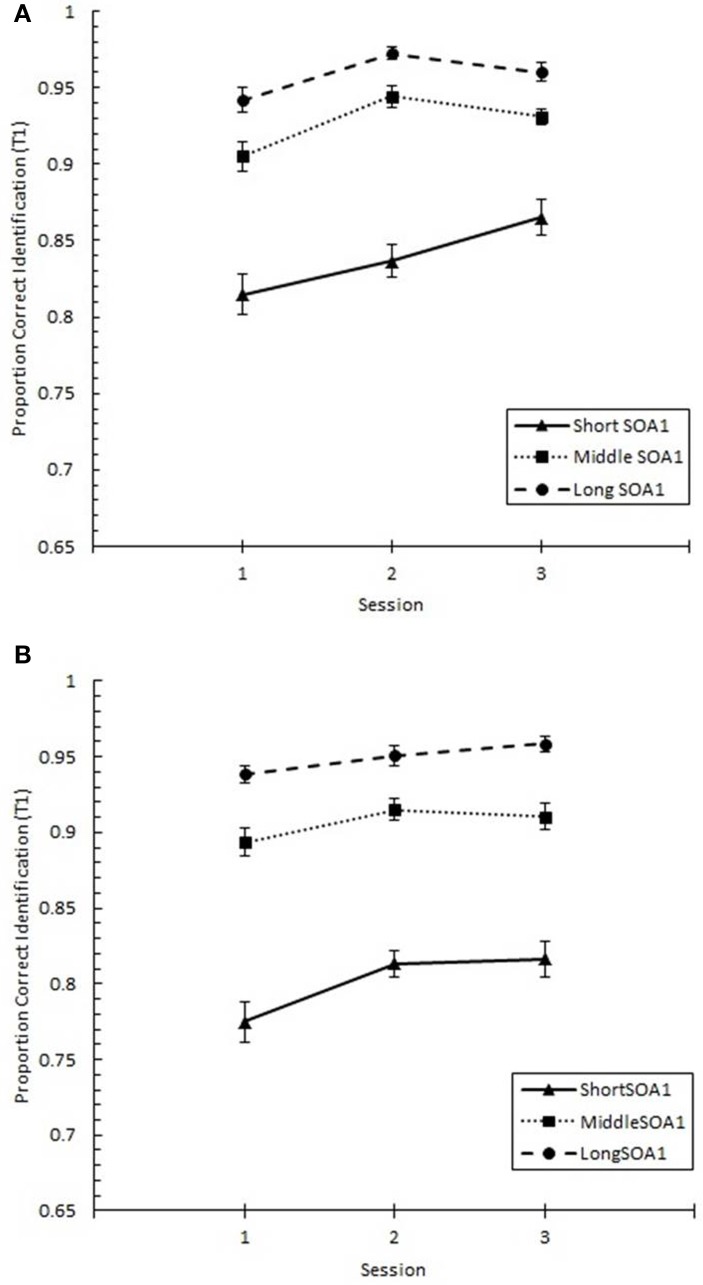
**Proportion correct first target identification (***p***(T1)) at Lag 1 as a function of Session for the random (A) and blocked (B) conditions**. *p*(T1) at Lag 1 is plotted separately for the short SOA1 (70 ms, triangles), middle SOA1 (98 ms, squares), and long SOA1 (126 ms, circles).

**Figure 9:**
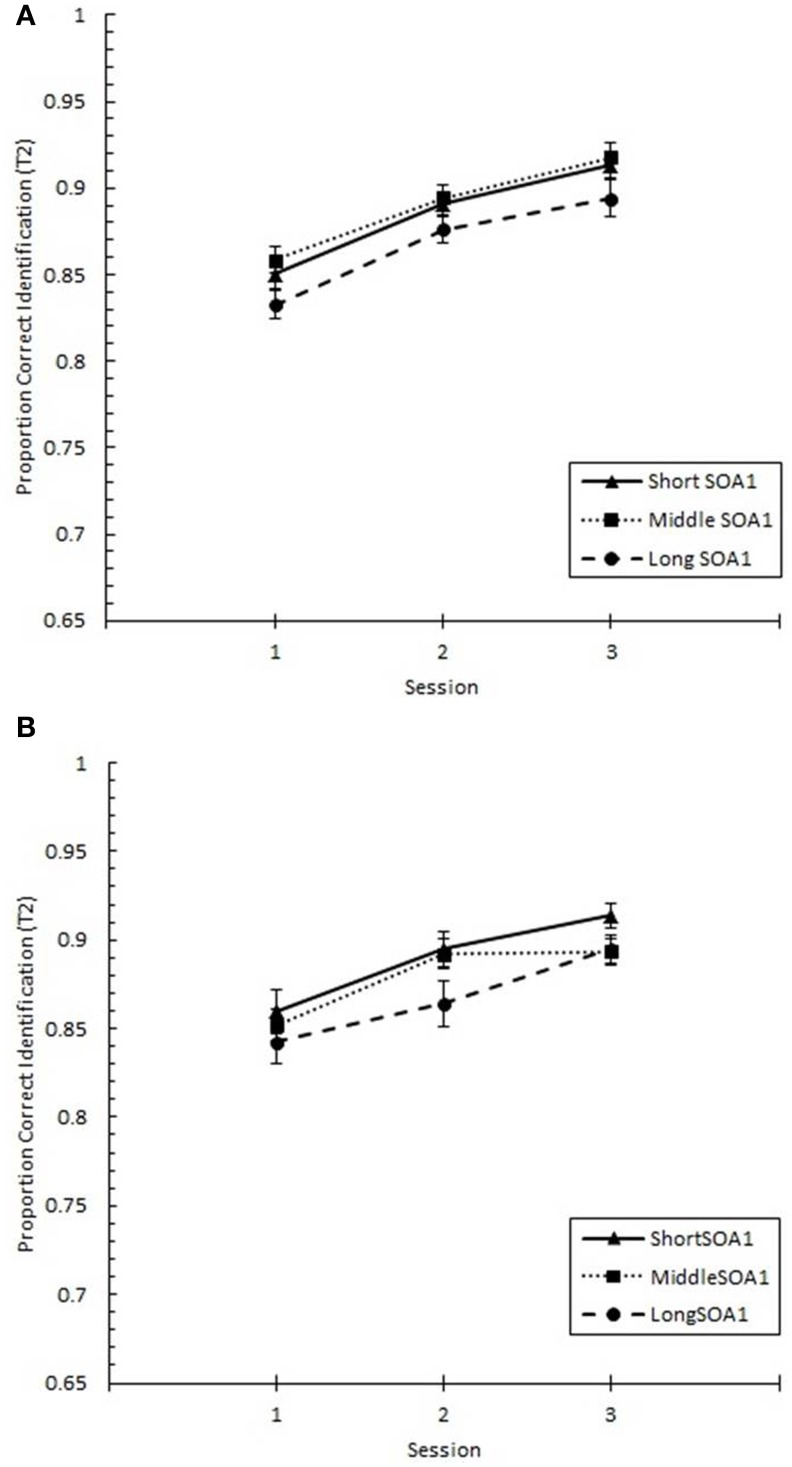
**Proportion correct second target identification (***p***(T2)) at Lag 1 plotted as a function of Session for the random (A) and blocked conditions (B)**. *p*(T2) at Lag 1 is plotted separately for the short SOA1 (70 ms, triangles), middle SOA1 (98 ms, squares), and long SOA1 (126 ms, circles).

Focusing on Lag 1, we conducted a 3 (Session) × 3 (SOA1) × 2 (SOA1 Consistency) ANOVA on *p*(T1), shown in Figure 8, and on *p*(T2), shown in Figure 9. Both increased with practice, *Fs* > 12, *ps* < 0.0001. As predicted, *p*(T2) was greater the shorter the SOA1, *F*_(2, 90)_ = 3.43, *p* < 0.05, and the opposite was true for *p*(T1), *F*_(2, 90)_ = 138.24, *p* < 0.0001. No other effects reached statistical significance, *p*s > 0.09.

**Figure 10:**
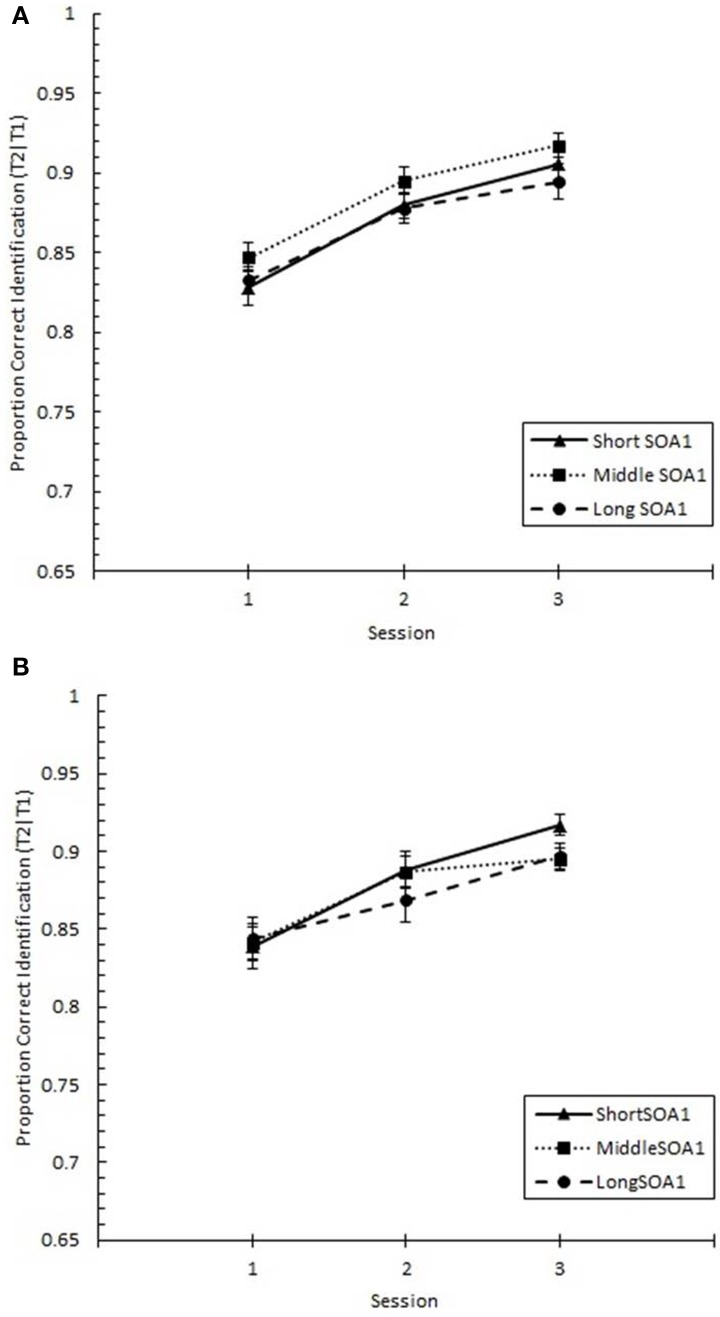
**Proportion correct second target identification conditional on correct first target identification (***p***(T2|T1)) at Lag 1 (lag 1 sparing) plotted as a function of Session and for the random (A) and blocked conditions (B)**. Lag 1 sparing is plotted separately for the short SOA1 (70 ms, triangles), middle SOA1 (98 ms, squares), and long SOA1 (126 ms, circles).

The same analysis conducted on Lag 1 sparing (*p*(T2|T1) at Lag 1), shown in Figure 10, revealed significant increases with practice, *F*_(2, 90)_ = 28.17, *p* < 0.0001. However, no other effects were statistically reliable, *p*s > 0.2.

#### Reversal errors

**Figure 11:**
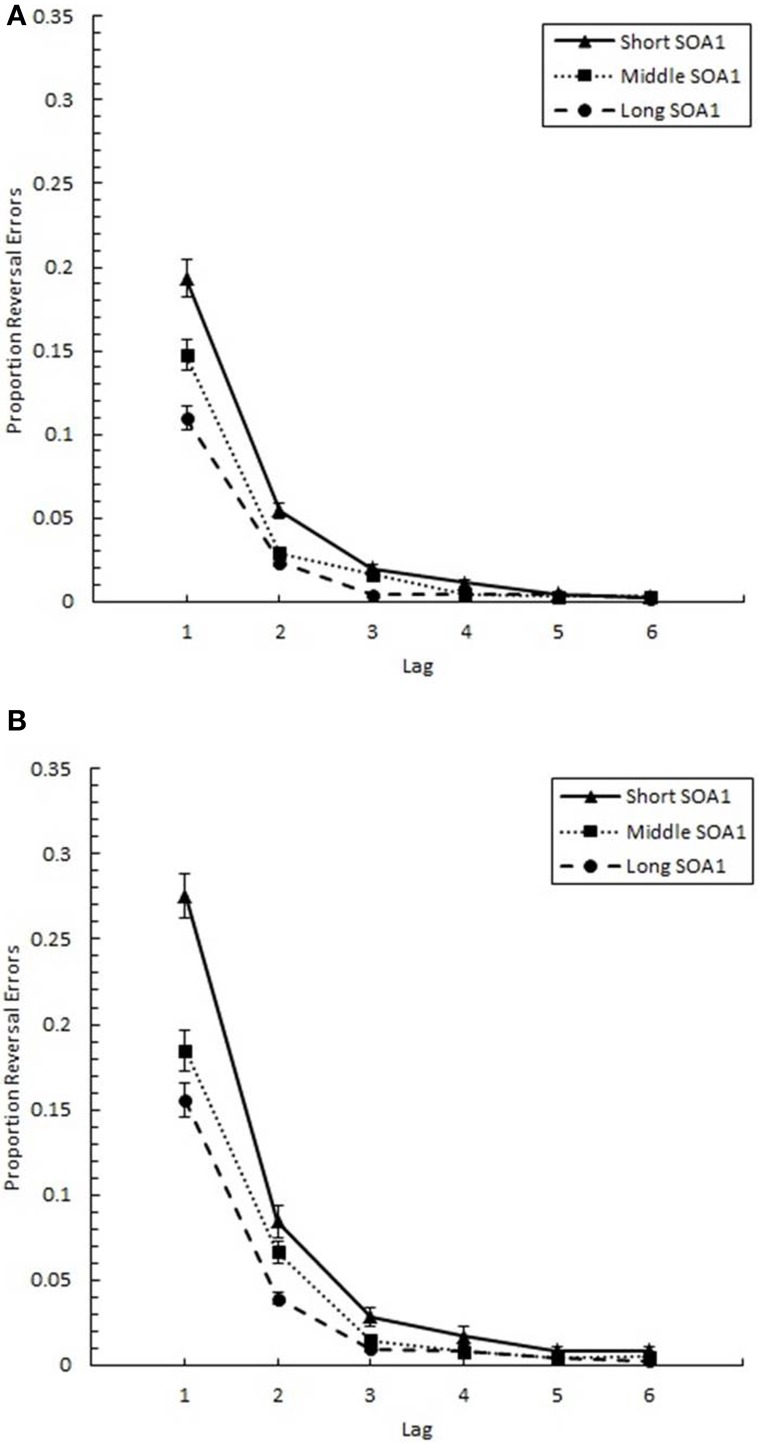
**Proportion reversal errors (***p***(reversal)) plotted as a function of Lag for the random (A) and blocked conditions (B)**. *p*(reversal) is plotted separately for the short SOA1 (70 ms, triangles), middle SOA1 (98 ms, squares), and long SOA1 (126 ms, circles).

The proportion of reversal errors for those trials where both targets were correctly identified (*p*(reversal)), shown in Figure 11, was analyzed in a 6 (Lag) × 3 (SOA1) × 2 (SOA1 Consistency) ANOVA. This analysis revealed a significant main effect of Lag on *p*(reversal), *F*_(5, 225)_ = 198.13, *p* < 0.0001. A Scheffé's test indicated *p*(reversal) was greatest at Lag 1 (*M* = 0.185), followed by Lag 2 (*M* = 0.055), which, in turn, showed greater *p*(reversal) than the other lags (*M* = 0.010). As expected, the main effect of SOA1 was also significant, *F*_(2, 90)_ = 48.29, *p* < 0.0001. A Scheffé's test showed that *p*(reversal) was significantly greater at the short SOA1 (*M* = 0.064), than at the middle (*M* = 0.043), and long (*M* = 0.032) SOA1s. The Lag × SOA1 interaction was significant, *F*_(10, 450)_ = 23.43, *p* < 0.0001; SOA1 main effects were found only at Lags 1–4, *F*s > 5, *p*s < 0.01.

As predicted, more reversal errors were made in the blocked than in the random condition, [*F*_(1, 45)_ = 4.90, *p* < 0.05]. Also, the Lag × SOA1 Consistency interaction was significant, *F*_(5, 225)_ = 5.39, *p* < 0.001; significant differences between the blocked and random conditions were only found at Lags 1 and 2, *F*s > 5, *p*s < 0.05.

**Figure 12:**
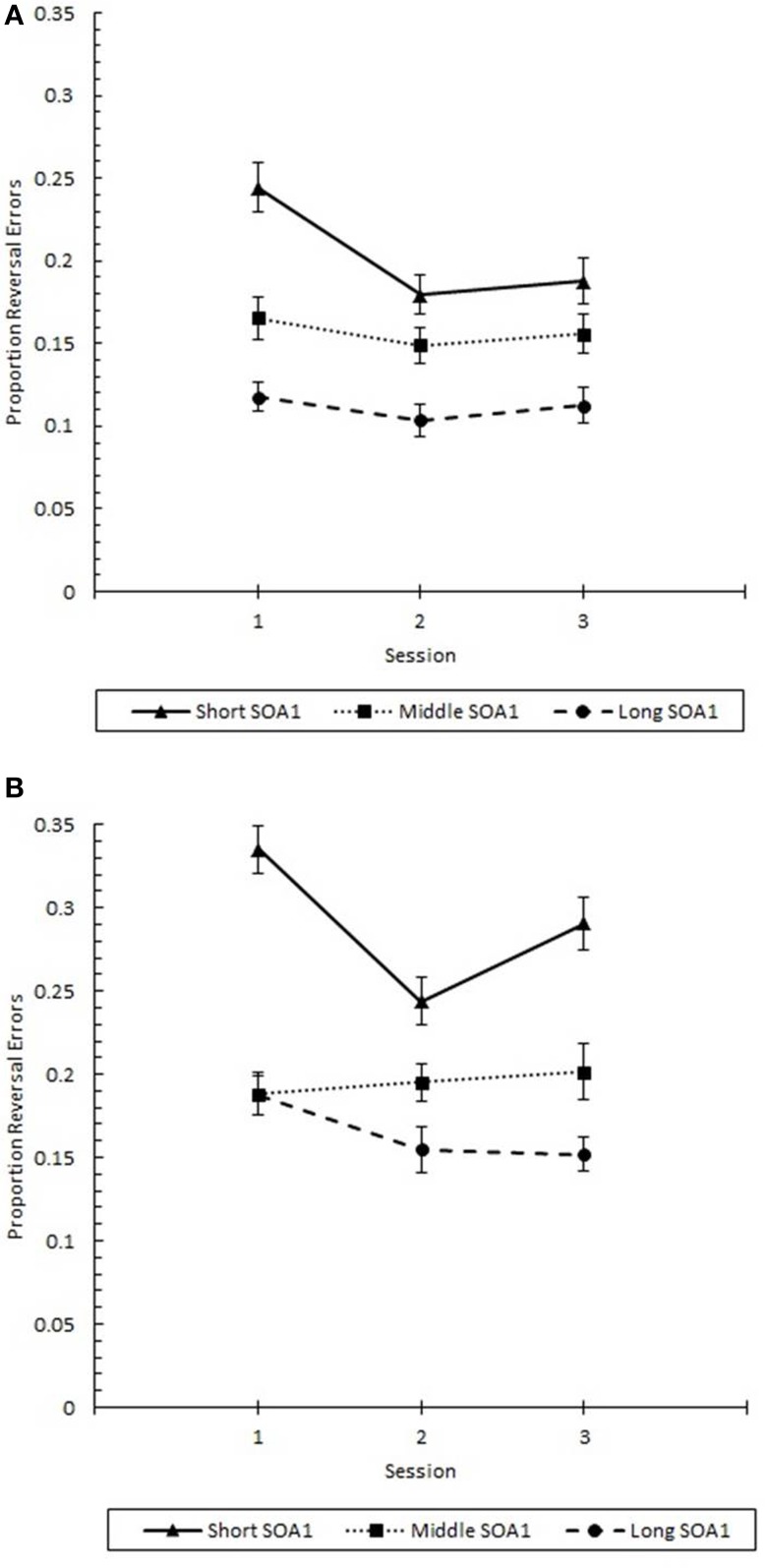
**Proportion reversal errors (***p***(reversal)) for Lag 1 trials plotted as a function of Session for the random (A) and blocked conditions (B)**. *p*(reversal) is plotted separately for the short SOA1 (70 ms, triangles), middle SOA1 (98 ms, squares), and long SOA1 (126 ms, circles).

Finally, the effects of practice (Figure 12) were examined in a 3 (Session) × 3 (SOA1) × 2 (SOA1 Consistency) ANOVA on *p*(reversal) at Lag 1. Consistent with the analysis above, *p*(reversal) was greater in the blocked condition (0.217) than in the random condition (0.157), *F*_(1, 45)_ = 5.79, *p* < 0.05. Also, *p*(reversal) was greater the shorter the SOA1, *F*_(2, 90)_ = 33.79, *p* < 0.0001. *p*(reversal) showed a continuous and linear decrease from session to session, *F*_(2, 90)_ = 4.04, *p* < 0.05. In addition, the differences among SOA1 tended to be reduced with practice, *F*_(4, 180)_ = 2.18, *p* < 0.07.

## Discussion

We investigated whether implicit learning of timing could lead to attentional modulation that facilitated a relatively high level cognitive process—letter identification—in an RSVP task. We manipulated the consistency of target timing independently of lag and target duration in a two-target RSVP series. The results suggest that information about target timing was learned implicitly and that this learning improved attentional control. In addition, we observed the effects of extended practice on RSVP performance. Over the course of the 3-day practice period, a clear and continuous pattern of improvement in performance was found both in terms of target identification rates and AB magnitude.

### The implicit learning and control of temporal attention

We manipulated a subtle time constraint in order to minimize the potential for participants to become aware of a timing manipulation and react in a strategic manner, allowing us to attribute changes in performance to implicit temporal learning. Despite the subtlety of the SOA1 manipulation, our results showed that implicit learning occurred for the consistent temporal constraint in a way that influenced attentional control. First, the magnitude of the AB deficit was reduced with practice, and this practice-related decrement in AB was greater when SOA1s were consistent in the blocked condition than in the random condition. Second, consistent SOA1s led to a greater proportion of target reversal errors.

Previous research showed that performance could be enhanced through explicit knowledge about timing through pre-cues in single stimulus trials (Karlin, 1959; Westheimer and Ley, 1996; Coull and Nobre, 1998; Grosjean et al., 2001; Kristjánsson et al., 2010) and in RSVP tasks (Martens and Johnson, 2005; Hilkenmeier and Scharlau, 2010; Visser et al., 2014) through explicit instructions about temporal consistency (Tang et al., 2014; Visser et al., 2014) or fixed TOAs implemented between participants (Willems et al., 2015). In contrast, evidence was lacking that performance could be influenced by implicitly learned information about a fixed time constraint in the RSVP paradigm.

Our study extends the research exploring enhancements in temporal attentional control brought about through the manipulation of lag probabilities. In an auditory rapid serial presentation task requiring the detection of two targets, Shen and Alain (2012) manipulated the probabilities of T2 lags (2 or 8) to be high (80%) or low (20%). They found that detection rates improved for both targets when T2 was displayed at the high probability lag, suggesting that expectations about lag led to fine-tuned temporal control of attention rather than a gross shift in attentional resources from T1 to T2. Willems et al. (2015) found that a moderate amount of practice with consistent T2 timing (450 trials) led to temporal expectations. In that study, blocking target lag between participants led to enhanced T2 identification and a reduced AB.

These studies observed attentional modulations arising from explicit expectations about lag. In contrast, our study is unique in providing strong evidence for implicit learning of subtle time constraints that were independent of lag. Interestingly, our results contrast with Martens and Johnson (2005, Experiment 1), where evidence for learning was lacking with more salient time differences. It is possible that the extended amount of practice in the current study provided more opportunity for implicit temporal learning to emerge. In addition, fixing the position of T1 might have improved our chances of observing the effects of temporal learning on attention. Tang et al. (2014) found that displaying T1 at a fixed position (position 2) in an RSVP series magnified the performance benefits of attending to T2.

These findings are congruent with the idea that consistent timing of targets following T1 facilitated the encoding of T2. The shortened duration of T2 processing can lead to reduced competition with T1 for perceptual or memory processing resources during the AB interval. In addition, when T1 and T2 are both identified in Lag 1 trials, two-stage models of AB, such as the simultaneous type serial token models (Chun and Potter, 1995; Bowman and Wyble, 2007; Wyble et al., 2009) assume enhanced T2 processing efficiency can increase the proportion of trials where T2 is encoded faster to working memory relative to T1, resulting in increased target reversal errors. T2 processing might be facilitated through temporal learning through more optimal timing of an attentional pulse (Reeves and Sperling, 1986; Shih and Sperling, 2002) in terms of its latency and precision (Vul et al., 2008). More precise temporal control through modified attentional dynamics might occur for either target. Physiological changes related to temporal expectations were found by Shen and Alain (2011). In that study, T2-related P3b from the midline parietal area increased in amplitude and decreased in latency accompanied positive behavioral changes induced by instructions to attend to specific lags. Interestingly, Willems et al. (2015) found behavioral changes resulting from temporal expectations were accompanied by changes in attentional dynamics related to T1, gauged through pupil dilation dynamics. Specifically, practice with consistent T2 timing resulted in earlier attentional peaking to T1, and successful T2 identification was associated with lower T1 related attentional pulses. A similar change in attentional dynamics to T1 may have occurred in the current study, which, like Willems et al. (2015), had T1 position fixed across trials. Contrary to previous work on AB reduction, in the Willems et al. (2015) study, RSVP practice did not influence the time course of T2 related attentional peaking, although successful T2 identification was associated with earlier and higher attentional peaking. Perhaps, stronger effects of temporal consistency on attentional dynamics could be found with a greater number of practice trials. Both the Shen and Alain (2011) and Willems et al. (2015) studies are thought to have involved explicit temporal learning and conscious attentional changes. Therefore, future studies mapping detailed attentional dynamics for implicit temporal learning would be necessary to compare the nature of attentional adjustments between explicit and implicit learning situations.

Although the increase in reversal errors with consistent timing in our study could be explained by improved temporal attentional selection of T2, an alternative explanation should also be considered. That is, the probability of encoding both targets into a common perceptual episode could have increased. Akyürek et al. (2012) showed that integrated encoding accounted for a large proportion of order reversal errors in RSVP tasks. Expected stimulus duration is one factor that can influence the likelihood of integrated encoding. Akyürek et al. (2008) found increased target reversal errors with a longer (70 ms) rather than shorter (30 ms) expected stimulus duration, possibly due to a slower perceived rate of stimulus presentation. An expectation for the short stimulus duration was associated with electroencephalographic activity indicating increased likelihood of separate T1 and T2 encoding relative to an expectation for the long stimulus duration (Akyürek et al., 2007b). Although it was not likely that participants were consciously aware of the different SOA1s in the current study, it is possible that consistent timing altered the perception of overall stimulus presentation speed relative to random timing.

Integrated target encoding could be increased by faster perceptual processing of T2, a lengthening of the episodic encoding time window, or both. Although, the idea that consistent timing might allow T2 to be processed faster is reasonable, the idea that consistent timing might encourage a more relaxed time course of memory processes that admits T2 into the same encoding process with T1 also remains plausible. Thus, the results of our study do not allow us to discriminate between the *T2 facilitation* and *encoding lengthening* possibilities. Also, our results cannot distinguish between the prior entry (Hilkenmeier et al., 2012) and integration (Akyürek et al., 2007b) explanations. Indeed, it is conceivable that any combination of mechanisms contributed to the effects of timing consistency on reversal errors.

Since the SOA1s were positioned within the first 250 ms of the onset of the first RSVP stimulus, it is important to note that our results supporting implicit temporal learning concern subtle time intervals that occur early in an RSVP stream, where *attentional awakening* is underway (Ariga and Yokosawa, 2008). Thus, future research would be needed to explore whether such implicit learning could take place throughout different positions in an RSVP series.

At first glance our results comparing blocked and random SOA1 conditions contrast with those of Martin et al. (2011), where temporal irregularity of stimulus presentation led to improved identification for T1 and T2 and reduced AB relative to regular timing. In that study, irregular timing consisted of *ISI*s that varied by 17–153 ms. The relative deviation from the regular timing condition of 85 ms ISIs was far greater than the current study, and it is possible that this led to conscious awareness of the temporal irregularity. This interpretation is in line with the explanation provided by Martin et al. (2011), where temporal noise in distractor presentation was thought to enhance attentional processing to target-irrelevant information, resulting in an attenuation of attentional overinvestment in T1 (Olivers and Meeter, 2008). Such changes in T1 directed attention were thought to improve T2 identification and reduce AB. Such benefits of irregular timing may be weak or absent in the current study due to the subtle nature of the time intervals involved and the implicit nature of the temporal learning that was induced in this study.

The effects of temporal consistency discussed up to this point appear to be independent of specific effects of SOA1 on performance, as SOA1 Consistency did not interact with SOA1 in any of the measures we examined. Shorter SOA1s led to less T1 identification and greater T2 identification, especially at Lag 1. Similar trade-offs between T1 and T2 at very short target onset asynchronies demonstrates the vulnerability of T1 to strong competition from T2 for attentional resources (Potter et al., 2002) and backward masking from subsequent stimuli. The relationship between backward masking and AB was somewhat complex. Although (Ouimet and Jolicoeur, 2007) and Visser (2007) found that difficulty in T1 identification due to backward masking inflates AB, at least initially, the shortest SOA1 led to worst T1 identification but lowest AB Magnitude. However, practice led to a decrease in both SOA1 effects on T1 and AB Magnitude, congruent with those expectations. Also, our result showing practice related reductions for AB only for longer SOA1s suggest that backward masking might limit the extent of AB reduction that can be achieved through practice.

### Power law improvement and the development of attentional skill

To our knowledge, this is the first study to systematically investigate how the amount of practice influences performance in an RSVP task. We found that the identification rates of both targets improved continuously over 3 days of practice and more than 1500 trials. In addition, a large portion of the improvement was concentrated on the AB time interval, leading to the reduction of the AB. Each of these measures showed substantial improvements that showed diminishing returns with practice, that is, as a negatively accelerating (power) function of practice. Not surprisingly, because T1 identification was near ceiling throughout practice, it showed only a modest rate of improvement. However, the power function improvement found for T2 identification cannot be attributed to ceiling effects, since this measure never approached maximal performance. This shape of the learning curve is characteristic of a wide range of perceptual-motor and cognitive skills and considered a defining feature of skill acquisition (Newell and Rosenbloom, 1981). These results demonstrate the ability of participants to maximize the goals of the RSVP task through practice.

This research expands previous work pointing to positive effects of practice on RSVP performance. Practice led to a reduction in the AB deficit in RSVP tasks requiring T1 identification and T2 detection (Maki and Padmanabhan, 1994; Nakatani et al., 2012), but only when target and distractor sets were consistent; AB was not significantly reduced when target and distractor sets were variable (Maki and Padmanabhan, 1994). Thus, the critical role of target set consistency in attentional search may apply to serial visual presentations as well as simultaneous visual presentations (Schneider and Shiffrin, 1977; Shiffrin and Schneider, 1977). Spatial consistency may also be crucial for practice related benefits in RSVP performance. Braun (1998) failed to find evidence of improvement after thousands of practice trials, perhaps due to varying the spatial location of target presentation. It is worth pointing out that the above studies showing benefits of practice utilized tasks where T1 and T2 were processed to meet different task goals. In such cases, it remains difficult to gauge the extent to which improvements in RSVP performance resulted from enhanced attentional selection or from improved skill in task switching from identification to detection. Several studies reported practice related AB reductions in RSVP tasks that required identification of both T1 and T2 (Martens and Johnson, 2005; Olivers and Nieuwenhuis, 2005; Slagter et al., 2007; Livesey et al., 2009; Taatgen et al., 2009; Choi et al., 2012). These studies involved attentional manipulations designed to draw attention to T2, such as displaying T2 in a salient color (Choi et al., 2012), making T2 predictive of a cue for a secondary task (Livesey et al., 2009), or providing an explicit cue that predicts the position of T2 in the RSVP series (Martens and Johnson, 2005, Experiments 2 and 3). In others, the distribution of attention to the RSVP task was manipulated using concurrent tasks (Olivers and Nieuwenhuis, 2005; Taatgen et al., 2009), meditation training (Slagter et al., 2007) and videogaming (Green and Bavelier, 2003 with a T1 identification and T2 detection task).

Our study is unique in revealing a *spontaneous* improvement in RSVP performance for all of our measures. Furthermore, these practice related changes occurred regardless of whether timing was consistent in the blocked condition or random. Presumably, the improvement we found in T1 and T2 identification was not caused by an increase in the total amount of attentional resources across the entire period of practice, since there is no a priori reason to assume that attentional resources increased continuously over the 3-day experimental period. A more viable explanation would focus on an enhancement in the efficiency of attentional processes. The speeding up of component processes in a complex task can account for “power law” improvement in many skills (Rosenbloom and Newell, 1986; Logan, 1988; Compton and Logan, 1991). The practice related improvements we found can be attributed to changes in attention that result from learning the consistencies in the way stimuli were presented rather than strategic shifts in attentional deployment that can be induced by enhancing stimulus salience, including target-distractor sets and spatial consistency. In addition, the temporal consistency inherent in periodic stimulus presentation might have served to induce a stable mode of attentional dynamics that enhanced the effects of practice on RSVP performance.

In previous studies, the simultaneous improvement in the identification of both targets was found to be accompanied by neurophysiological changes signifying enhanced efficiency in attentional selection. Nakatani et al. (2012) found such behavioral changes were associated with a diminished T1-evoked P3 amplitude, which indicated increased efficiency for spatial attentional selection. Similarly, Slagter et al. (2007) found AB reduction to be associated with a diminished T1-evoked P3 for participants who engaged in meditation training, a manipulation thought to modulate attentional distribution in space and time. The enhanced efficiency in T1 processing indicated by the changes in these two studies can free up attentional resources to be reallocated to aid T2 processing, which itself can become more efficient (Nakatani et al., 2012). Although neurophysiological measurements were not employed in the current study, our results are consistent with such neural changes.

The massive reduction in AB demonstrated here is congruent with theories that explain AB as a side effect of excess control processes intended to protect T1 memory consolidation (Olivers and Meeter, 2008; Taatgen et al., 2009). However, because total elimination of AB was not found, our results do not contradict stage-based or other bottleneck theories of AB with built in architectural constraints that directly constrain the successful cognitive processing of rapidly presented stimuli (Raymond et al., 1992; Chun and Potter, 1995; Jolicoeur, 1998; Di Lollo et al., 2005; Kawahara et al., 2006; Akyürek et al., 2007a; Bowman and Wyble, 2007). Whether continued practice over a longer period of time would result in further improvement in target identification, or even total AB elimination, is a question for future research.

### Adaptive processes in individual participants and the flexibility of temporal attention

**Figure 13:**
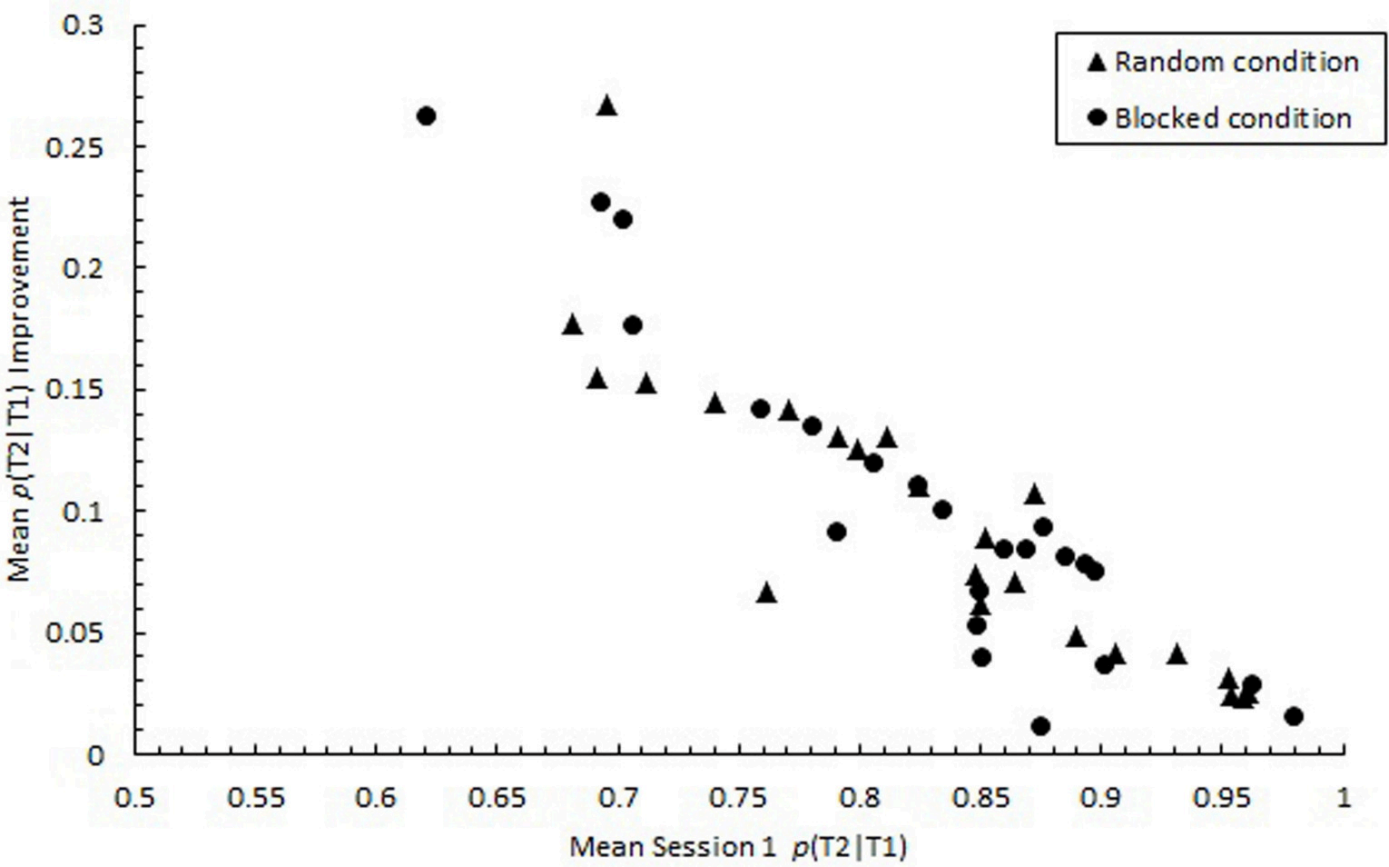
**Participant means of improvement in proportion correct second target identification conditional on correct first target identification (***p***(T2|T1)) computed as mean ***p***(T2|T1) in Session 3 minus mean ***p***(T2|T1) in Session 1**. Mean *p*(T2|T1) improvement is plotted as a function of mean *p*(T2|T1) in Session 1 for the random (triangles) and blocked conditions (circles).

Consistent with studies that highlighted individual differences in RSVP performance (Martens et al., 2006; Taatgen et al., 2009), *p*(T2|T1) varied substantially among individuals during the initial practice session, ranging from 0.283 to 0.979, *SD* = 0.383. However, by the final practice session, the variability among individuals decreased considerably, with mean *p*(T2|T1) ranging from 0.656 to 0.996, *SD* = 0.262. As illustrated in Figure 13, improvement in performance was greatest for those who initially performed the poorest. A negative correlation was found between Session 1 performance and the degree of improvement in performance from Session 1 to Session 3 for both the random, *r*_(21)_ = −0.89, *p* < 0.0001 and blocked conditions, *r*_(21)_ = −0.93, *p* < 0.0001. Thus, the 3-day practice period appeared to neutralize individual differences in attentional processes that were apparent initially. Thus, individual differences in AB found in previous work and initially in the current study may not represent inherent differences in the ability to control attention.

Our results showcase the general success with which participants developed skill in achieving the RSVP task goals. The adaptive attentional responses to the subtle changes in timing as well as other sources of consistent information in our study demonstrate the sensitivity of the attentional system to environmental information. Like motoric skills whose force, timing, and spatial movements are adjusted to the goals of bodily actions, attentional control itself appears to be a mental skill that becomes fine-tuned to the goals of a cognitive task. The approach taken here opens the door to novel questions concerning the opportunities and boundaries in acquiring attentional skill.

## Author contributions

JS designed the study, and SC and YC acquired and analyzed the data. All authors contributed to the interpretation of results and writing of the manuscript.

## Funding

This work was supported by the National Research Foundation of Korea Grant from the Korean Government (NRF-2006-2005112).

### Conflict of interest statement

The authors declare that the research was conducted in the absence of any commercial or financial relationships that could be construed as a potential conflict of interest.
